# Death of Dr. J. B. Smith

**Published:** 1865

**Authors:** Robert R. McIlvaine, Chas. P. Wilson

**Affiliations:** Hall of Academy of Medicine


### DEATH OF DR. J. B. SMITH.

HALL OF ACADEMY OF MEDICINE,

May 13, 1865.

"At a meeting of the Academy of Medicine, held this afternoon, to adopt measures expressive of sorrow at the death of Dr. J. B. Smith, the following preamble and resolutions were, after appropriate remarks, passed:

WHEREAS: In the all-wise providence of God, our brother and fellow-member, Dr. Joseph Byrd Smith, has been removed to another world, we, the members of the Cincinnati Academy of Medicine, mourn his loss, while we cherish his many virtues. For eighteen years a general practitioner of medicine in this city, he has won the regard of all by his high professional deportment. No man had a higher regard for truth. In his intercourse with us, as well as his patients, his fine sense of justice, with his great generosity, especially characterized all his acts. He was devoted to his profession, and permitted nothing to interfere with his attention to his patients.

As an observer and a student, few surpassed him. As a devoted member of this society, we shall miss him greatly. His place will not soon be filled. In his social relations he was a kind father and loving husband, and a faithful friend. He was especially marked for a strong practical judgment, which characterized him in his large practice. His charity was very large, manifested by years of attentive service to many poor, distressed families. His modesty was a prominent feature. No man disliked *pretence* and *sham* more than he did.

He was a loyal man to his country, and in the last two years has performed an immense amount of labor as surgeon in charge of Washington Park Hospital.

He died with the Christian's hope.

*Resolved*, That in the death of Dr. J. B. Smith, the Cincinnati Academy of Medicine has lost a distinguished member—a gentleman devoted to a science, whose first and only object is the relief of humanity.

*Resolved*, That in his decease the profession at large has lost a valuable member—a man who, in his quiet life, illustrated the true principles of the good physician, a charitable disposition, a high-toned behavior, and an abiding love for truth.

*Resolved*, That the members of this Academy deeply sympathise with the family of our deceased brother, and that a copy of these resolutions, signed by the Chairman and Secretary, be transmitted to them.

ROBERT R. MCILVAINE, *President.*

CHAS. P. WILSON, *Secretary.*

The above, which we copy from one of our city papers, contains the action of the "Academy of Medicine" in reference to the death of one of our most prominent physicians. Dr. J. B. Smith, as all know who knew him, was one of the most active progressive men in the medical profession in this city. He was a man of more than ordinary talent, and that coupled with industry, energy, and perseverance, made him what he was. He occupied an elevated and commanding position, without even the appearance of assumption. He was the commander of his own position. His professional attainments were of an extensive range. Though he was decisive, firm and commanding, he was always respected, and had no enemies. Wherever he formed attachments they were true and ardent in an eminent degree; we know this from personal experience. He has occupied various responsible positions, and always with honor to himself, and profit to others. He has more than once been a medical teacher, and has almost from the beginning of his medical career been connected to a greater or less extent with the hospitals and infirmaries of this city. He was a teacher in the Ohio College of Dental Surgery for a number of years, occupying the chair of physiology and pathology; he was a superior teacher. Those throughout the country who have been his pupils will mourn his loss, for none knew him but to love him. As a patriot, a friend, a husband and father he was preeminent; and above all we doubt not he was a Christian.

But he is gone and those who knew him will not soon forget him, for he was of a mould that made an impress whenever he touched. To his family will his name ever be a joy and an honor.

